# Treatment of Solitary Painful Osseous Metastases with Radiotherapy, Cryoablation or Combined Therapy: Propensity Matching Analysis in 175 Patients

**DOI:** 10.1371/journal.pone.0129021

**Published:** 2015-06-23

**Authors:** Mario Di Staso, Giovanni Luca Gravina, Luigi Zugaro, Pierluigi Bonfili, Lorenzo Gregori, Pietro Franzese, Francesco Marampon, Francesca Vittorini, Roberto Moro, Vincenzo Tombolini, Ernesto Di Cesare, Carlo Masciocchi

**Affiliations:** 1 Department of Biotechnological and Applied Clinical Sciences, Division of Radiation Oncology and Radiobiology, S. Salvatore Hospital, University of L'Aquila, Medical School, L’Aquila, Italy; 2 Department of Biotechnological and Applied Clinical Sciences, Division of Radiology, S. Salvatore Hospital, University of L'Aquila, Medical School, L’Aquila, Italy; 3 Department of Radiological, Oncological and Anatomo-pathological Sciences, "Sapienza" University, Rome, Italy; 4 Service of Medical Physics, San Salvatore Hospital, L’Aquila, Italy; Taipei Medical University, TAIWAN

## Abstract

**Purpose:**

aim of this study was to identify outcomes in pain relief and quality of life in patients with a solitary painful osseous metastasis treated by radiotherapy, cryoablation or the combination using a propensity score matching study design.

**Materials and Methods:**

175 patients with painful bone metastases were included in the study. Twenty-five of them underwent a radiation course (20 Gy in five daily fractions) 15 days after the cryoablation. These subjects were retrospectively matched by propensity analysis with a group of subjects treated by radiotherapy (125 subjects) and with a group treated byCryoablation (25 subjects). The pain relief in terms of complete response, rate of subjects requiring analgesics after treatments and the changes in self-rated quality of life were measured. Informed consent was obtained from the subject and the study was approved by the local Ethical Committee.

**Results:**

An higher proportion of subjects treated by cryoablation (32%) or cryoablation followed by RT (72%;) experienced a complete response compared with patients treated by radiotherapy alone (11.2%). After Bonferroni correction strategy, the addition of radiotherapy to cryoablation significantly improved the rate of complete response compared with cryoablation alone (p = 0.011) and this paralleled with an improved self-rated quality of life. Seventeen subjects (13.6%) of patients in the radiotherapy group, 9 (36%) in the cryoablation group, and 19 (76)% in the cryoablation- radiotherapy group did not require narcotic medications.

**Conclusions:**

The addition of radiotherapy to cryoablation favorably impacts on perceived pain, with a favorable toxicity profile. However, our data should be interpreted with caution and could serve as a framework around which to design future trials.

## Introduction

Bone metastases are the main cause of cancer-associated death in patients with cancer [1] and represent one of the most common sites of metastasis [2–3].Breast, prostate, and lung cancers collectively represent the primary tumors that more frequently metastasize to bone [4]. Furthermore, considering that a reduced bone matrix is found in patients suffering from breast and prostate cancers treated with hormonal therapies, bone metastasis grows into a compartment bone that is itself already weakened [5–6].

Among the potential complications related to bone metastasis, pain is the symptom most frequently referred by patients [4] resulting in a drastic deterioration of their quality of life (QoL) [1, 4, 5, 7, 8].For patients with overt bone metastases, current treatment objectives are designed to decrease tumor burden, reduce skeletal-related events and maximize pain control [5]. Current management of skeletal metastasis includes pain management/analgesia, systemic therapy, radiation therapy (RT), surgery, and ablative techniques [9]. However, the long-term-results of many of these treatment modalities may be not fully satisfying [10,11].

Thermoablation is a family of non-surgical approaches used to treat otherwise unrespectable tumors and using different power sources. Various delivery methods of ablation exist. They include radiofrequency ablation (RFA), microwave ablation, high intensity focused ultrasound (HIFU), laser ablation, and cryoablation (CA) [12–14]. These techniques, initially developed for patients with malignant primary or metastatic liver tumors, have proven to be useful therapeutic options for the management of bone tumors [15–18]. However, these ablative approaches have rarely been used in combination in with RT [18]. Recently has been demonstrated that the association of RFA with RT was well tolerated, with a satisfactory profile of adverse events. [18–20]. In this study, for the first time, we investigated whether the sequential addition of RT to CA could favorably affect clinical management of painful bone metastatic lesions compared with CA and RT delivered as individual treatments.

## Materials and Methods

### Patient selection

In this study, patients older than 18 years with radiological and histological confirmed painful solitary bone metastases were prospectively selected to undergo CA procedures followed by RT. Magnetic resonance imaging (MRI) examination, computed tomography (CT), and nuclear isotope (technetium 99) bone scan were planned within the 4 weeks prior to the procedures. Other eligibility criteria included: (i) a pain score of 5 or more on the validated visual analog scale (VAS) over the prior 24 hours (or a score of less than 5 with the use of narcotic medications); (ii) pain localized to the site of the bone metastases; (iii) life expectancy of greater than 3 months; (iv) and Karnofsky performance status (KPS) score of greater than 70. Exclusion criteria were: (i) a painful area previously treated with RT or palliative surgery; (ii) radiographic evidence of spinal cord or cauda-equina compression; (iii) lesions positioned within 0.5 cm from a critical structure such as the spinal cord, brain, aorta, inferior vena cava, bowel, or bladder; (iv) abnormal fracture of the treatment site.

The combined treatment was as arranged as follows: CA followed by RT 15 days later when technically feasible. Between September 2011, and April 2014, 41 consecutive patients receiving CA followed by RT were treated. Sixteen out of 41 subjects (39%) were excluded since (i) refused to participate in the study (3/41; 7%), (ii) they did not satisfy the inclusion criteria (5/41; 12.2%) and (ii) they did not match with any subject included in the groups treated by CA or RT after propensity analysis (8/41; 19.5%). The group of patients undergoing the CA-RT was retrospectively compared and matched by propensity analysis with a group of subjects treated with RT or CA. The subjects included in RT or CA groups were retrospectively selected from all who were treated in the Radiotherapy and Interventional Radiology units, respectively. Analgesic consumption of all patients was recorded. Written informed consent was obtained from the subject and the study was approved by the San Salvatore Hospital IRB (Deliberation n. 89/2012)

### Treatment procedures

RT was delivered by three-dimensional conformal technique with a dose of 20 Gy in five fractions of 4 Gy over 1 week. Percutaneous cryotherapy ablation was carried out under conscious sedation with an argon-based cryotherapy system (SEEDNET GOLD Cryoablation System, Galil Medical LTD, Israel). After sterile preparation, one or more cryoprobes (ICEROD, Galil Medical LTD, Israel) were introduced into the target lesion by CT guidance by experienced radiologists. The cryoprobes were introduced in a parallel arrangement approximately 2 cm apart. For larger lesions, a cluster of cryoprobes was placed within 1 cm of the tumor margin to provide adequate coverage of the outer border of the target lesion. Cryoprobe positioning was confirmed by CT imaging. Rapid freezing of the target lesion (-100°C within a few seconds) was obtained and two 15-minute freezes separated by a 10-minute thaw were used at each cryoprobe position. At the end of each procedure, CT was performed to ensure that the extent of ablation was confined to the target tissue and that there was no substantial damage in the tissue surrounding the target. After all combined procedures, patients were observed for 2 hours in the recovery room and then were admitted to the hospital for a minimum of 24 hours.

### Patient assessment

A full physical examination was performed and data on direct and indirect changes in pain levels were assessed by VAS and medication level questionnaire. QoL was assessed with a single question from the McGill Quality of Life Questionnaire (MQOL) [21].The strongest evidence of validity comes from comparison with the single-item quality of life measure [21].

### Study endpoints and response criteria

The primary endpoints were the percentage of patients with a (1) complete response (CR) at 12 weeks after treatments as previously described [22,23]. The secondary endpoints were (1) the rate of subjects requiring analgesics at 12 weeks after treatments and (2) the changes in self-experienced QoL. Complete response was defined as a pain score of 0 at treated site with no concomitant increase in analgesic intake (stable or reducing analgesics in daily oral morphine equivalent [OMED]). Partial response was defined as pain reduction of 2 or more at the treated site on a scale of 0 to 10 scale without analgesic increase, or analgesic reduction of 25% or more from baseline without an increase in pain. Pain progression was defined as increase in pain score of 2 or more above baseline at the treated site with stable OMED, or an increase of 25% or more in OMED compared with baseline with the pain score stable or 1 point above baseline. Stable-intermediate response was defined as any response that is not captured by the complete response, partial response, or pain progression definitions.

### Statistical analysis

The data analyzed in this report were derived from a population-based observational study. In order to reduce treatment selection bias and determine realistically the treatment effects, a case control-matched propensity analysis was performed. Pairwise nearest neighbor matching with a caliper was used to minimized distance within matched sets applying a caliper of 0.1 on the propensity score scale. Increasing the number of controls included in each matched set may increase the precision of the estimated treatment effect. The optimum number of controls needed to match to each treated subject may range from 1 to 5. Increasing the number of controls matched to each treated subject will increase the size of the matched sample, resulting in estimates of treatment effect with increased precision. Multivariate logistic regression was used to calculate the predicted probability of the dependent variables as well as the propensity score for all observations in the data set. The dependent variables included in the multivariate analysis were age, KPS, primary tumors, anatomic site, VAS scale, and QoL before procedures. A 1:5 matched analysis was performed where one case (subject treated by CA or CA followed by RT) was matched to five controls (subjects treated by RT).

The primary endpoint of this feasibility study was that, for patients with painful bone metastasis, pain relief achieved following CA-RT in terms of CR should be higher than that achieved following RT alone. The current study was powered to determine an increase of 21% or greater in the CR at 12 weeks after CA-RT with respect to RT alone. The literature indicates that from 11 to 21% of intention-to-treat (ITT) patients achieved CR after RT [24]. Thus, we set the rate of CR after RT at 11% (P0 = 11%). Using a two-sided test and a 5% type I error adjusted for Bonferroni correction (p<0.0166), with the matched control to case ratio of 1:5, 25 subjects in the experimental groups (CA group and CA-RT group) and 125 in the control group (RT) would provide greater than 80% power to detect an increase of 21% (P1 = 32%).

Continuous variables not normally distributed (Shapiro-Wilk test) were presented as medians and 95% confidence intervals (95%CI). The Mann-Whitney U test was used to evaluate the difference between two groups and Kruskal-Wallis test was used to evaluate the difference among more than two tests. If the Kruskal-Wallis test was statistically significant, apairwise comparison of subgroups was performed according to Conover. Dichotomous variables were summarized by absolute and/or relative frequencies. The chi-squared test or Fisher’s exact test was used to evaluate the difference between two groups. For multiple comparisons, the alpha value threshold was adjusted by using Bonferroni correction. All tests were two-sided except where specified and were determined by Monte Carlo significance. An alpha value threshold of 0.05 was used. All statistical analyses were performed using the SPSS statistical analysis software package, version 10.0 (IBM Corporation 1 New Orchard Road, Armonk, New York 10504–1722 United States).

## Results

### Local pain control and self-perceived QoL

Table 1 lists the clinical and demographic characteristics of treated patients stratified according to propensity score and treatment received. All subjects were valuable at the end point of 12 weeks. This was mainly due to the selection criteria (solitary bone lesion) which configure this group of patients as long survivors. A higher proportion of subjects treated by CA (32% [8/25]; p = 0.018) or by CA followed by RT (72% [18/25]; p<0.0001) experienced a CR at 12 weeks with respect to patients treated by RT alone (11.2% [14/125]; Table 2). Interestingly, the addition of RT to CA significantly improved the rate of CR with respect to CA used as individual treatment (p = 0.011). A PR was more frequently observed in patients treated by RT (42.4% [53/125]) or CA (36% [9/25]) with respect to subjects treated by CA followed by RT (12% [3/25]) (Table 2).

**Table 1:** Clinical characteristics according propensity score.

Characteristics	RT (n = 125)	CA-RT (n = 25)	CA (n = 25)	p value
Age, Y*	68 (66 to 69)	69 (65 to 71)	67.5 (64.4 to 70.6)	0. 0.454°
VAS Scale*	7 (6 to 7)	7 (6 to 8)	7.5 (5 to 7.6)	0.766°
**Sex, No (%)**				0.950°°
***Male***	61 (48.8)	13(52)	12(48)	
***Famale***	64 (51.2)	12 (48)	13 (52)	
**KPS, No**				0.908°*
***91–100***	64 (51.2)	12 (48)	11 (44)	
***70–89***	61 (48.8)	13 (52)	14 (56)	
**Tumor Size, cm (longest diameter)**	4 (4 to 5)	5 (4 to 5)	4 (3.4 to 6)	0.099°
**Primary Tumors, No (%)**				0.940°*
***Lung Cancer***	38 (30.4)	6 (24)	6 (24)	
***Prostate Cancer***	41 (32.8)	8 (32)	8 (32)	
***Renal Cancer***	9 (7.2)	2 (8)	4 (16)	
***Colorectal Cancer***	8 (6.4)	2 (8)	2 (8)	
***Breast Cancer***	29 (23.2)	7 (28)	5 (20)	
**Metastasis Location, No(%)**				0.961°*
***Pelvis***	52 (41.6)	9 (36)	8 (32)	
***Sacrum***	29 (23.2)	6 (24)	7 (28)	
***Rib***	10 (8)	2 (8)	2 (8)	
***Vertebrae***	22 (17.6)	4 (16)	4 (16)	
***Humerus***	9 (7.2)	2 (8)	2 (8)	
***Femur***	3 (2.4)	2 (8)	2 (8)	
**Characteristics**	**RT (n = 125)**	**CA-RT (n = 25)**	**CA (n = 25)**	pvalue
Medical Systemic Treatments **				
***Bisphosphonates***	35(28)	9 (36)	8 (32)	0.701°°
***Narcotic Analgesics***	125 (100)	25 (100)	25 (100)	1.0°*
***HormonalTherapy***	34 (27.2)	7 (28)	8 (32)	0.888°°
***Chemotherapy***	80 (64)	16 (64)	15 (60)	0.745°*
***Immunotherapy***	9 (7.2)	2 (8)	4 (12)	0.023°°

KPS = Karnofsky performance status

° Kruskal-Wallis test; Medians and CI95%

°* Chi Square test

°° Fisher’s Exact test; RT = radiotherapy; CA-RT = Cryoablation-Radiotherapy

**the sum of percentage in each group is over 100% since patients may perform more than one systemic treatment. In post hoc pairwise comparisons of subgroups the alpha error was set at 0.016 according to Bonferroni correction.

**Table 2:** Response rate following Radiotherapy vs Cryoablation vs Cryoablation combined with Radiotherapy at 12 weeks.

Response type No (%)	RT (N = 125)	CA(N = 25)	CA-RT(N = 25)	°Pairwise Comparisonsp value
**Complete response (No, %)**	14/125 (11.2)	8/25 (32)	18/25 (72)	CA vs RT p = 0.018CA vs CA-RT p = 0.011 CA-RT vs RT p<0.0001
**Partial Response (No,%)**	53/125 (42.4)	9/25 (36)	3/25 (12)	CA vs RT p = 0.711CA vs CA-RT p = 0.098 CA-RT vs RT p = 0.008
**Stable Pain or Progression (No,%)**	58/125 (46.4)	8/25 (32)	4/25 (16)	CA vs RT p = 0.270CA vs CA-RT p = 0.321CA-RT vs RT p = 0.009

**Fig 1:**
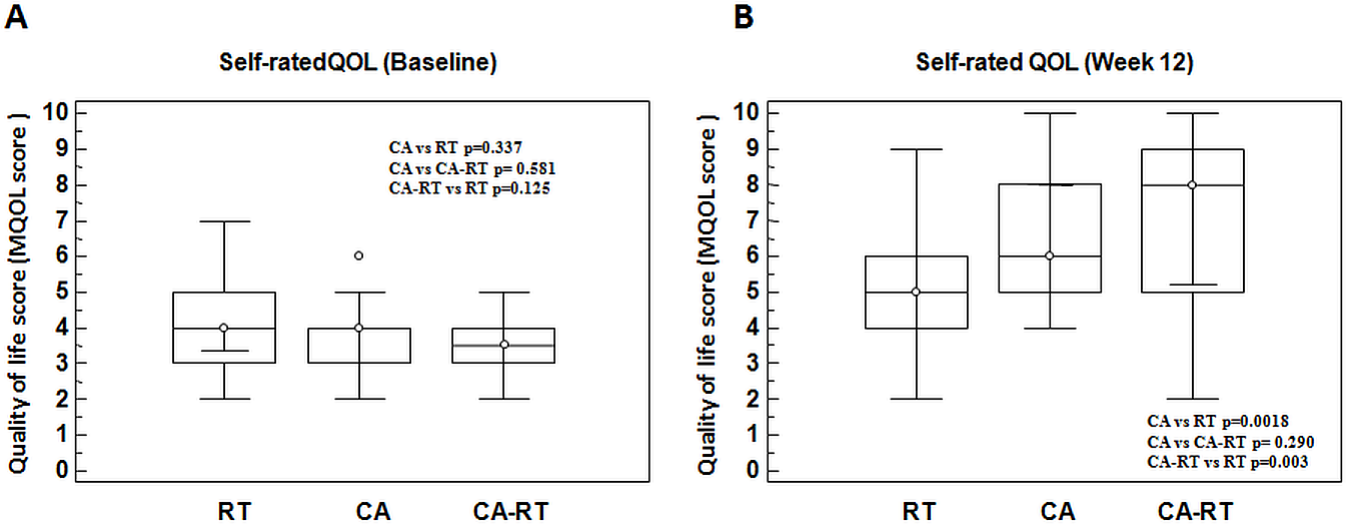
Quality of life score measured by single question from MQOL. Self ratedQoL before (A) and after treatments (B).

°Chi Square testor Fisher exact test. In post hoc pairwise comparisons of subgroups the alpha error was set at 0.016 according to Bonferroni correction; RT = radiotherapy; CA-RT = Cryoablation-Radiotherapy;

The higher rate of CR observed in patients treated with CA followed RT with respect to those treated with CA or RT paralleled an improved self-rated QoL (Fig 1). At 12 weeks, patients treated by CA followed by RT reported a higher improvement in self-rated QoL (MQOL score: 7, CI95% 5.4 to 9) with respect to subjects treated by RT alone (MQOL score: 5, CI95% 4 to 5). At the same time, patients treated by CA alone experienced a significant improvement of self-rated QoL (MQOL score: 6, CI95% 5 to 8) with respect to subjects treated by RT alone (MQOL score: 5, CI95% 4 to 5) (Fig 1). Finally, although an improvement in the self-rated QoL between CA and CA-RT was observed, the difference did not reach statistical significance (Fig 1).

All patients received oral narcotic analgesic over the month before treatment with CA, RT, or CA followed by RT (Table 1).In this regard, at 12 weeks, 13.6% of patients (17/125) in the RT group, 36% of patients (9/25) in the CA group, and 76% of patients (19/25) in the CA-RT group did not require narcotic medications (Table 3). This figures paralleled with the reduction in the 24-hours median morphine-equivalent dose. Before treatments the 24-hours median morphine-equivalent dose was 80 mg (95%CI 75–80) in the RT Group, 70 mg (95%CI 65–80) in the CA Group and 80 mg (CI95% 65.7–80) in the CA+RT Group with no significant difference among groups (p = 0.67). A significant decrease in the 24-hours median morphine-equivalent dose at week 12 was documented in the CA (p = 0.008) and CA-RT (p = 0.004) groups with respect to the RT group (Table 3). Overall, patients tolerated the CA and combined treatments well. Forty (80%) out of the 50 patients performed CA without complications. The complications were listed in Table 4.

**Table 3:** Post-treatment narcotic analgesic use and morphine equivalent dose at 12 weeks.

Narcotic medications	RT (N = 125)	CA(N = 25)	CA-RT(N = 25)	Pairwise Comparisonsp value
***None (No*, *%)Required (No*,*%)***	17 (13.6)108 (86.4)	9 (36)16 (64)	19 (76)6 (24)	°CA vs RT p = 0.016°CA vs CA-RT p = 0.010°CA-RT vs RT p<0.0001
**Morphine equivalent dose (mg)**	°*70 (60 to 80)	°*50 (2.9 to 60)	°*20 (2.2 to 50)	°°CA vs RT p = 0.008 °°CA vs CA-RT p = 0.71°°CA-RT vs RT p = 0.004

°Chi Square test; RT = radiotherapy; CA-RT = Cryoablation-Radiotherapy

°°Kruskal Wallis test with post hoc pairwise comparison of subgroups performed according to Conover

°* median and CI95%.

**Table 4:** Post cryoablation complications.

Number of Patients with complication	Tumor location	Complications	Clinical outcome and treatment
5/50 (10%)	Sacrum	Injury to encased sacral plexus	Gluteal, perineal, and thigh numbness resolved by corticosteroid treatment approximately 3 weeks after cryoablation
2/50 (4%)	Vertebrae	Transient injury to adjacent peripheral nerve	Resolved by corticosteroid treatment approximately 2 weeks after cryoablation
2/50 (4%)	Pelvis	Injury to encased sacral plexus	Gluteal, perineal, and thigh numbness resolved by corticosteroid treatment approximately 3 weeks after cryoablationInfection at the access site managed by percutaneous drainage and antibiotic treatment
1/50 (2%)	Humerus	Humerus fracture	Resolved by conservative approach

## Discussion

Radiation treatment is one of the most important non-surgical approaches for the management of osteolytic or osteosclerotic bone lesions [25] with sub-optimal overall response rate [25]. Interventional radiologists developed different ablative approaches, which demonstrated outstanding effects in terms of clinical efficacy in a wide range of clinical scenarios including bone metastatic disease. However, a number of patients may respond unsatisfactorily to these treatments in terms of pain control. Recently, the management of painful bone metastases was effectively achieved by combining ionizing radiation with RFA [18–20]. These encouraging preliminary results induced us to study if the addition of RT to CA would result in improved clinical efficacy. To our knowledge, no existing empirical study has addressed the question of whether percutaneous CT-based CA followed by RT achieves better pain control and improved self-rated QoL than CA or RT delivered as individual treatments in the management of osteolytic and/or mixed painful bone lesions. It is well-known that the localization of the bony metastases may affect the way subjects perceived their symptoms. This is an important methodological point since an heterogeneity the localization of the bony metastases may configure as a study bias. This bias was overcome by using propensity analysis and a balance among groups for this variable was achieved. Cryotherapy is a well-known ablative technique which, thanks to new miniaturized argon-gas devices, creates an “ice-ball” deep in bone, with less peri- and post-procedural pain. Callstrom and co-workers described the results of clinical trials in which percutaneous CA was used for the palliation of painful metastatic lesion [26–27]. Interestingly, they found that CA was an effective and safe way for palliation of pain related to metastatic lesions. Although authors did not specifically investigate this issue, they found that 62% of studied subjects received RT course prior to CA [27]. In these subjects no improvement in pain response with respect to subjects who did not perform RT was found [27]. In contrast, we found that when RT was delivered after CA, it favorably impacted on pain control in subjects with painful bone lesions. The main methodological difference with Callstrom’s study is that in ours, the radiation course was added sequentially to CA. The temporal sequence of two treatments was based on the hypothesis that RT may improve CA efficacy. Recent studies have reported achieving a synergistically increased rates of survival and tumor local control in both animal models and in patients with stage I lung cancer, when RFA was followed by RT [28–34]. We know that RT efficacy is largely dependent on tissue oxygenation, whereas this biological parameter does not influence CA efficacy. Bone lesions have a hypoxic microenvironment in their inner cores and CA, whose effectiveness is largely independent of tumor oxygen content, may effectively kill hypoxic centrally located tumor cells. In contrast, the outer ring contains fewer hypoxic tumor cells on which RT can effectively exert its cytotoxic effect. Additionally, it is possible that perturbation of the tumor microenvironment after ablation may improve the efficacy of subsequent RT on the malignant tissue [35]. However, the exact mechanisms through which CA followed by RT may relieve symptomatic pain remains largely unknown.

The pain experienced by subjects suffering from bone metastases may have several pathophysiologal mechanisms, including: (i) microfractures by direct metastatic bone invasion; (ii) periosteum distortion due to increased pressure on the endosteum; (iii) nerve-root compression or muscle spasm; and (iv) release of chemical mediators involved in the conduction of nociceptive impulses to the central nervous system [2,3]. The decrease of cancerous cell burden within bone tissue associated with a lowering of endosteum pressures and modification of the release of pain-related chemical mediators related with the combinational treatment may substantially influence the perception of pain intensity.

The improvement of CR rate we found, in terms of pain control, showed that 11.2% of subjects treated by RT achieved a CR. The rate of CR was significantly improved with respect to RT in subjects treated by CA (32%) and interestingly, when RT was sequentially added to CA, the percentage of patients achieving a CR was 72%. These data paralleled the improvement of self-rated QoL observed in subjects treated by CA and CA followed by RT with respect to subjects treated by RT alone. When RT was sequentially added to CA the subjects experienced improved QoL, although the difference did not reach statistical significance.

In terms of medical adjunctive treatment, all subjects took narcotic medications before treatments. At 12 weeks post treatment, 13.6% of patients in the RT group, 36% in the CA group, and 76% in the CA-RT group did not require narcotic medications and the 24-hour median morphine-equivalent dose significantly decreased with respect to baseline, with the major decrease found after CA or CA followed by RT. Finally, although the rate of complications we reported seems to be higher than that reported by Calstrom [20], the combinational treatment was well tolerated since 84% of patients were ablated without complications. The recorded complications were all transient and none of treaded subject presented irreversible injures at 12 weeks after treatments.

Several limitations affect our study. The main ones are the sample size and the use of a non-randomized study design. To date, large randomized controlled trials (RCT) have provided the strongest evidence for the efficacy of therapeutic procedures or treatments in the clinical setting. However, this bias has been mitigated by the use of a strategy based on propensity score analysis, which helped us to obtain groups of patients randomized post-hoc for important clinical characteristics. Thus, comparative analysis by propensity-matched pairs contributed to the results being less prone to methodological biases than other usual statistical methods. Finally, the positive results obtained in terms of both QoL and pain control may be partially imputable to the adjunctive oncological medical treatments that the subjects included in this study performed concomitantly with RT, CA and CA+RT. If these medical treatments have contributed to the effects measured in this study is unclear. In this regard, a strategy of assessment based on the measurement of outcome measures before the start of adjunctive oncological medical treatments may not ensure that the pain or the QoL perceived at that moment may be similar to that experienced when RT, CA or CA+RT was then effectively delivered. This situation is imputable to the fact that these medical treatments may go on for several weeks and during this time period the symptoms may profoundly change. However, we believe of having minimized this issue by adopting the propensity score analysis. In this regard the percentage of subjects using oncological systemic adjuvant treatments did not significantly differ across the groups making the confounding effect of this variable on outcome measure balanced in the groups. So, if we reasonably assume that at the time of local treatment with RT, CA or CA+RT the rate and the type of oncological medical treatments were comparable across the groups any improvement measured in pain or QoL was imputable to the aforementioned local treatments. Different may be the question concerning the fact if one or more of these oncological medical treatments may have potentiated the effects of RT, CA or CA+RT. It is very difficult to answer to this question since we cannot be sure which treatment is responsible for the enhancement of local treatments effects. All these reasons make our conclusions not equally generalizable to the population of patients suffering from solitary painful bone metastases who are both treated or not treated with multimodal oncological medical treatments. It is our opinion that the results here described are better suitable for the larger population of patients who is actively treated with multimodal oncological medical treatments.

Despite these methodological limitations our data seem to suggest, for the first time, that the addition of sequential RT to CA favorably impacts on the pain of painful bone metastases. However our results have to be interpreted with caution and to serve as a framework around which to design future large-scale RCT.
